# Interest of the Submental Flap in Facial Reconstruction

**DOI:** 10.7759/cureus.114291

**Published:** 2026-08-10

**Authors:** Boubacar Moussa Diadie, Buckat Hugues, Ayoub Bakhil, Amina Kahoul, Mouhamed R Ndoye, Lahcen Khalfi, Karim El Khatib

**Affiliations:** 1 Faculty of Medicine, Université Abdou Moumouni de Niamey, Niamey, NER; 2 Maxillofacial Surgery, Mohammed V Military Training Hospital, Rabat, MAR; 3 Department of Odonto-Stomatology and Maxillofacial Surgery, Omar Bongo Ondimba Military Teaching Hospital, Libreville, GAB; 4 Maxillofacial and Plastic Surgery, Mohammed V Military Training Hospital, Rabat, MAR; 5 Oral and Facial Surgery, Mohammed V Military Training Hospital, Rabat, MAR

**Keywords:** exercise, facial reconstruction, submental flap, surgery, tissue loss

## Abstract

The pedicled submental flap is a fasciocutaneous flap with an axial pedicle centered on the submental artery. It is indicated in maxillofacial surgery for intraoral tissue defects and cutaneous defects of the lower two-thirds of the face. Through careful analysis of case studies, significant anatomical variations according to patient age were highlighted, emphasizing the importance of considering this diversity when applying the technique. These anatomical findings improved the reliability of flap harvesting and enabled the development of new pedicle-lengthening methods to increase the arc of rotation. Its color match, texture, and simplicity of harvesting provide promising perspectives for this flap.

## Introduction

The submental flap is a valuable tool in facial reconstructive surgery. Inspired by platysmal flaps, it was conceived by Dominique Martin in 1990 [1]. Several studies have improved the reliability of its harvesting and increased its arc of rotation through pedicle-lengthening techniques such as venous Y-V plasty, retrograde harvesting, or hybrid forms [1].

The pedicled submental flap is indicated in maxillofacial surgery for intraoral tissue defects and cutaneous defects of the lower two-thirds of the face [2]. The submental flap is a myocutaneous island flap vascularized by the submental artery, a branch of the facial artery [3].

## Case presentation

A 55-year-old female patient with no significant medical history presented with a left centro-jugal lesion that had been evolving for approximately five years. Initially small, the lesion progressively enlarged and became ulcerated, prompting consultation in our department two months before treatment.

**Figure 1:**
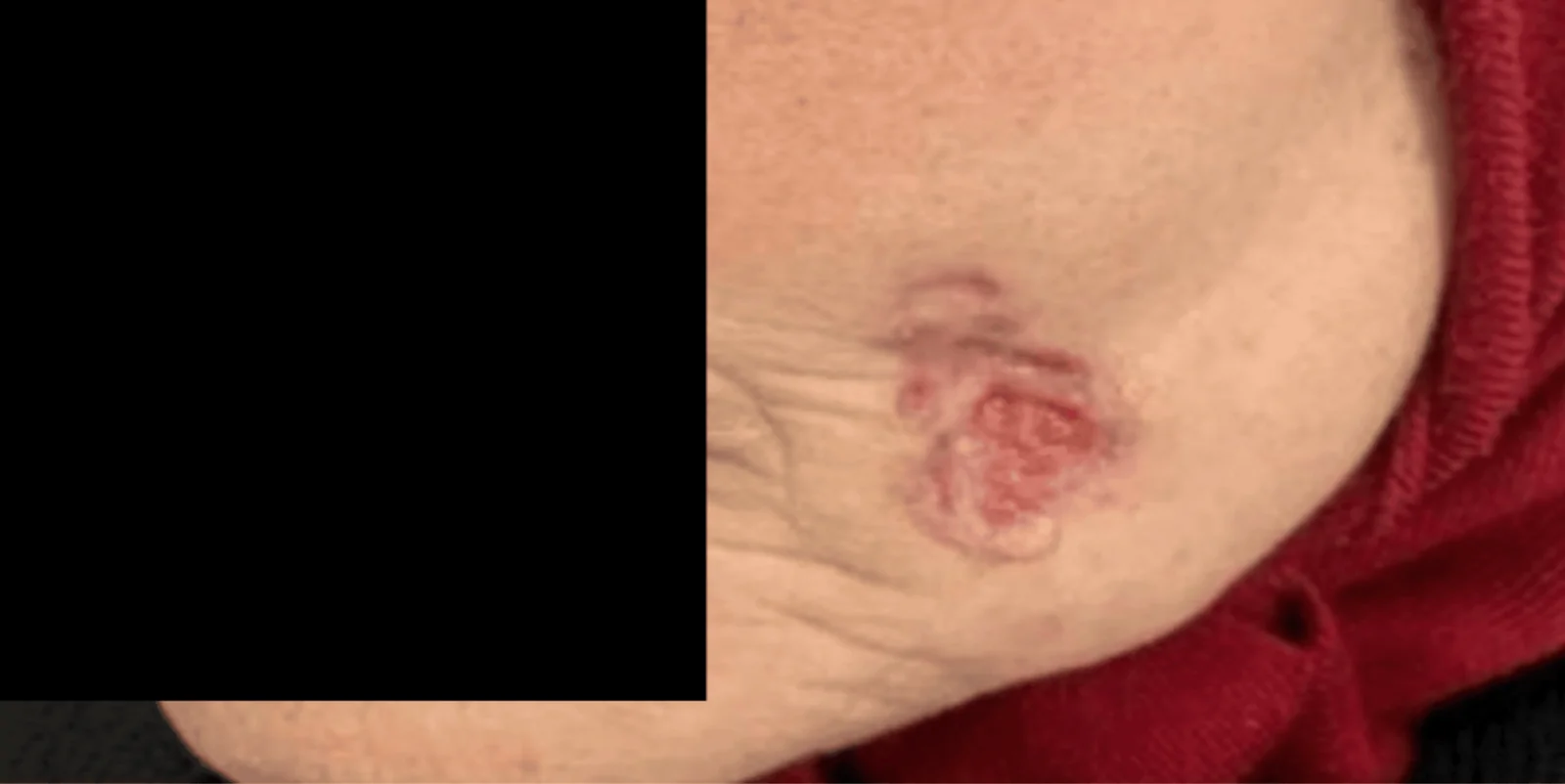
Preoperative photograph of the left centro-jugal ulcerative lesion

A skin biopsy was performed, and histopathological examination confirmed the diagnosis of basal cell carcinoma. The patient was therefore admitted for surgical management. Maxillofacial examination revealed an ulcerated and painful left centro-jugal lesion measuring approximately 3 cm in its greatest dimension, bleeding on contact, fixed to the superficial plane but mobile relative to the deep plane (Figure 1).

**Figure 2:**
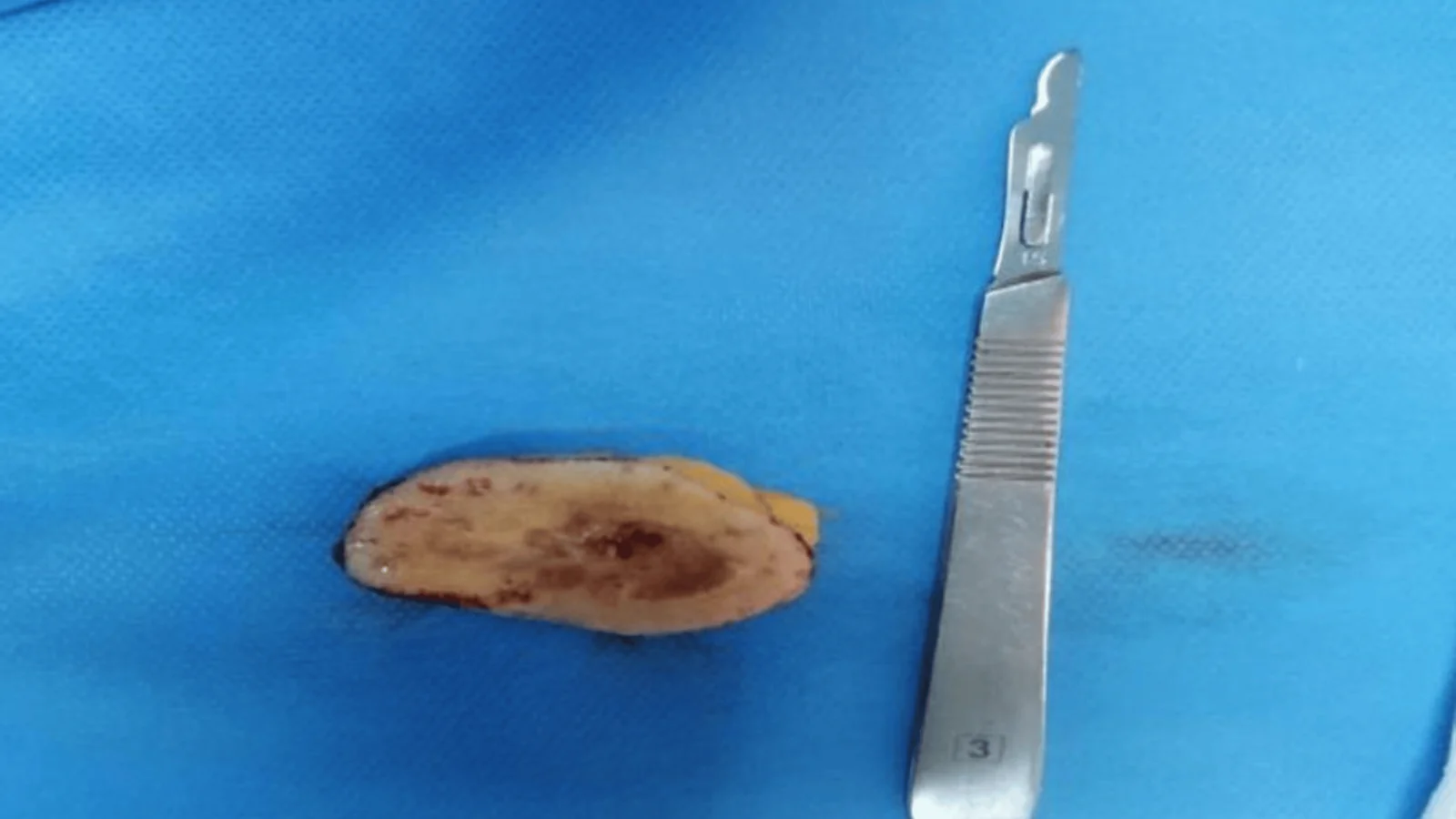
Photograph of the surgical specimen Excision of the cutaneous tumor with 1 cm margins

**Figure 3:**
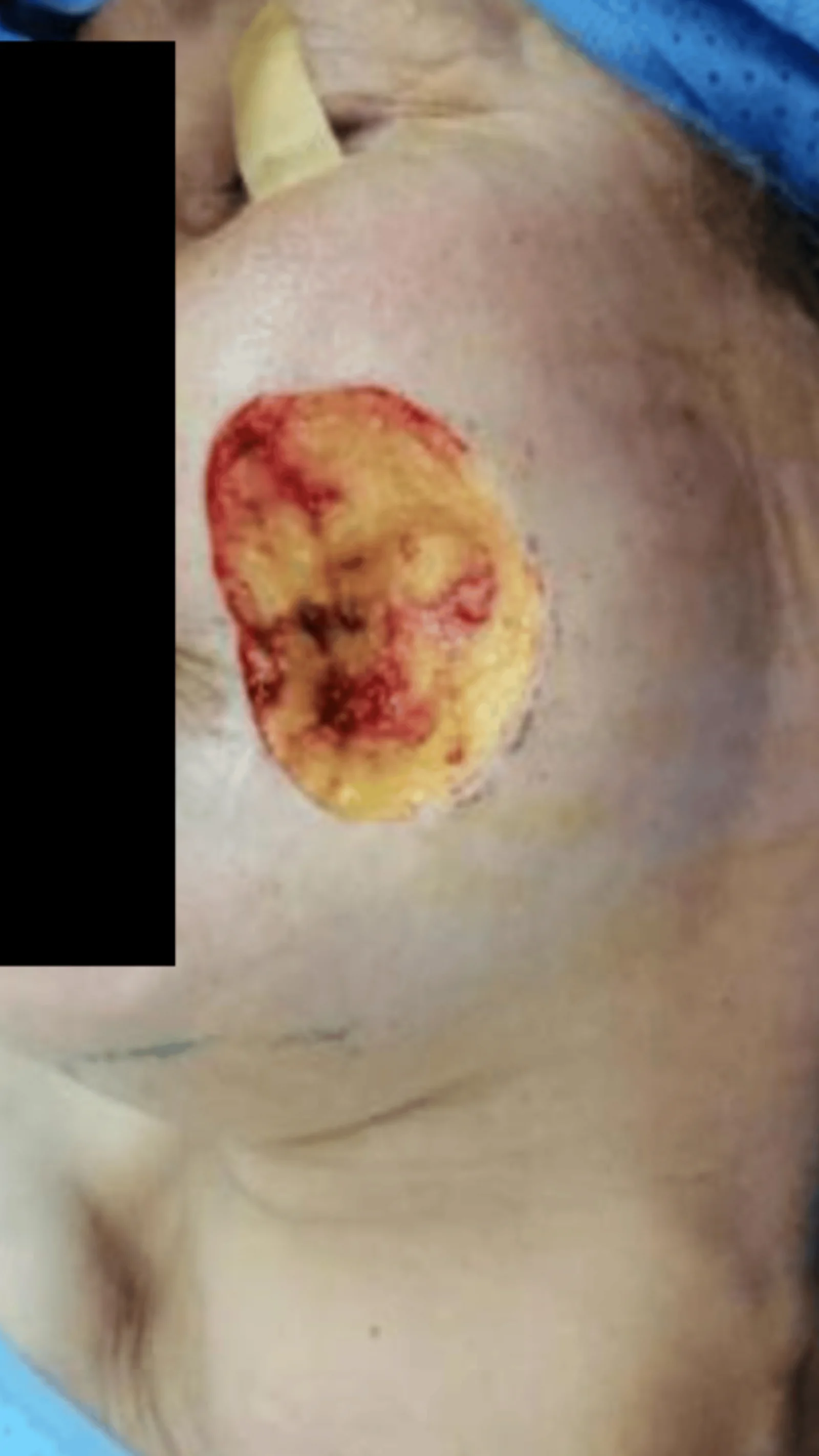
First intraoperative photo (tissue loss after tumor excision)

Facial sensitivity and motor function were preserved. Intraoral examination showed good mouth opening (interincisal distance greater than 4 cm), poor oral and dental condition, and no intraoral extension of the lesion. The openings of the Stensen and Wharton ducts were patent. Cervical examination revealed no palpable lymphadenopathy. The remainder of the physical examination was unremarkable. The patient underwent carcinologic excision of the skin lesion with 1 cm safety margins (Figure 2), resulting in an extensive jugal tissue defect requiring reconstruction (Figure 3). Cervical examination revealed no palpable lymphadenopathy. The remainder of the physical examination was unremarkable.

**Figure 4:**
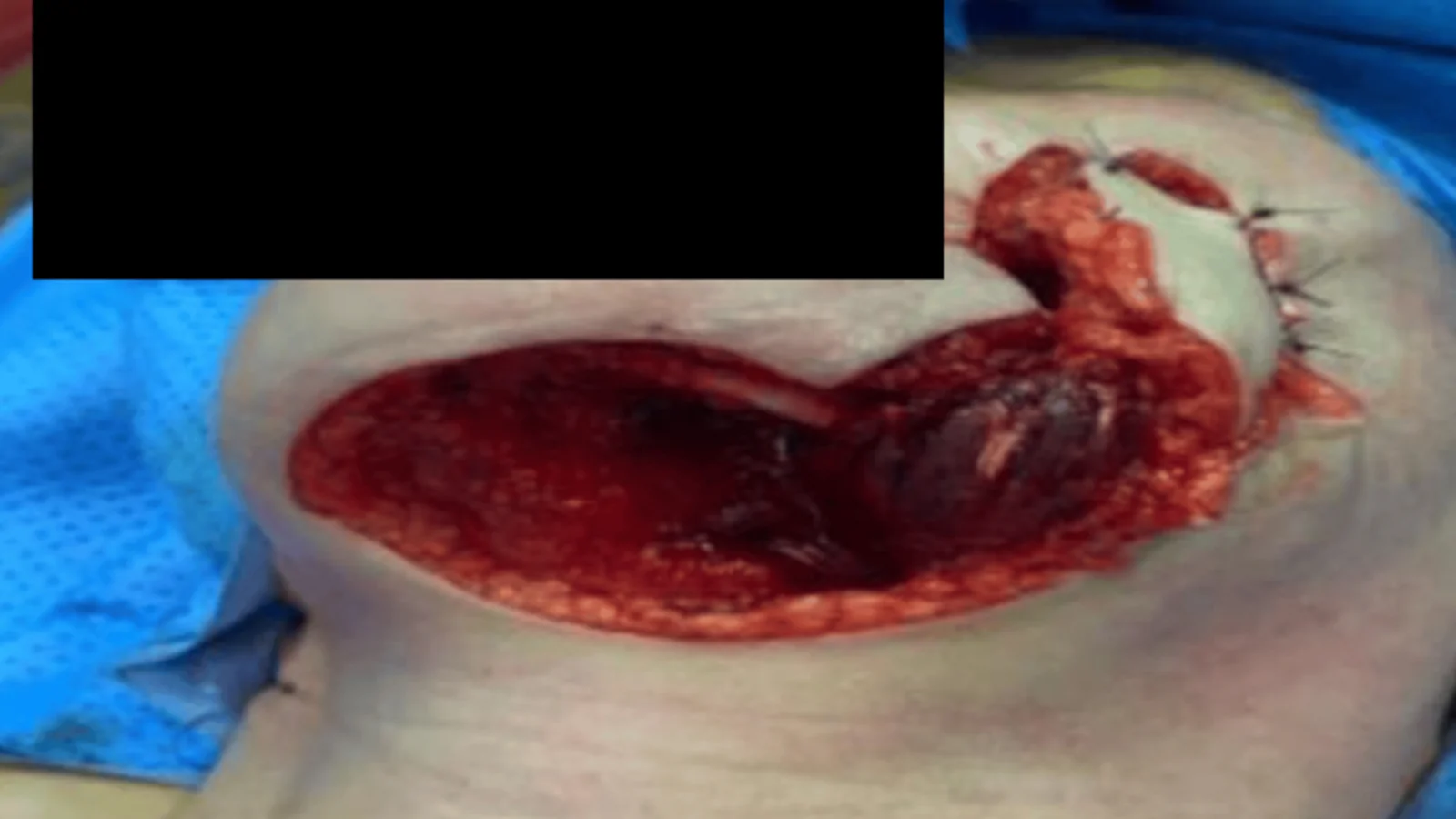
Second intraoperative photo (flap elevation)

**Figure 5:**
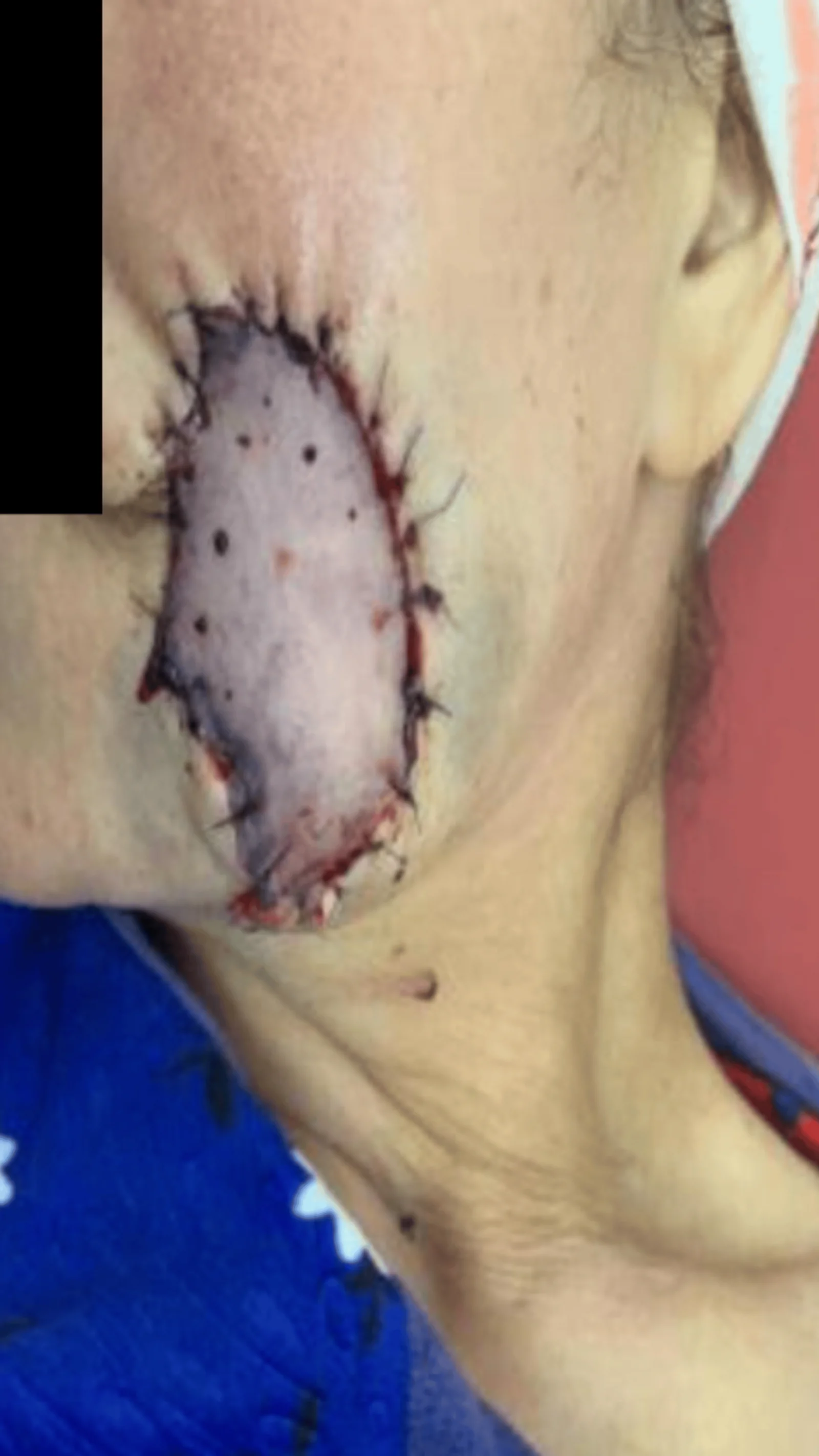
Photo after a few days (flap healing)

**Figure 6:**
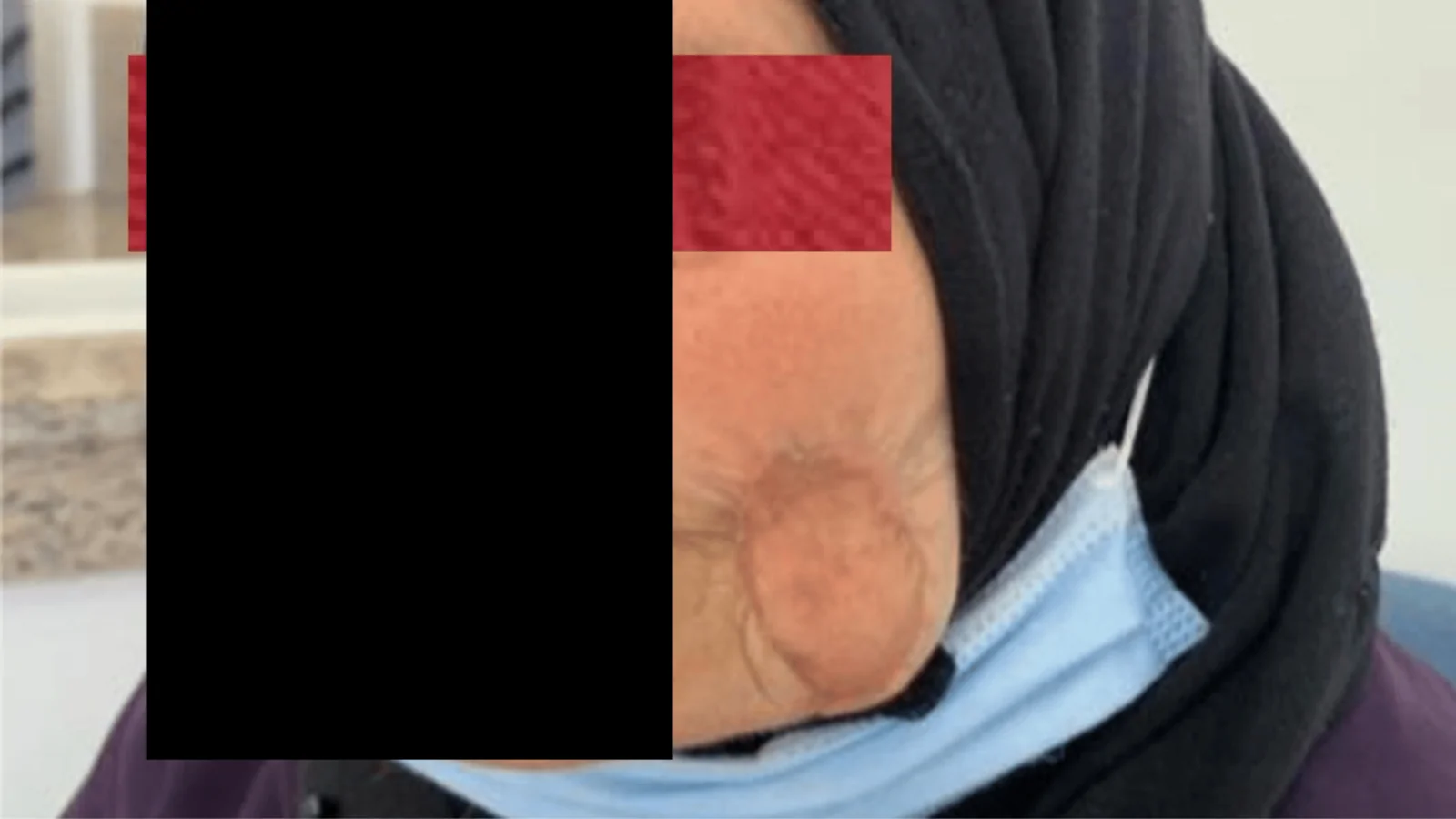
Photo after three months (good scar development)

An underlying tissue defect is observed after raising the flap; it was sutured without tension in two layers (Figure 4). Twenty-four hours after flap placement, a bluish appearance is visible, indicating minor compromise due to venous return issues (Figure 5). The patient was re-examined after three months: the flap had taken well, showing good healing (Figure 6). 

## Discussion

The pedicled submental flap currently represents a reliable and widely used reconstructive option in cervicofacial surgery, particularly for the management of tissue defects involving the lower two-thirds of the face and the oral cavity. Initially described as an extension of cervical flaps, it has undergone numerous technical improvements that have optimized its vascularization and expanded its arc of rotation [1,3].

From an anatomical perspective, this flap is vascularized by the submental artery, a branch of the facial artery, which provides excellent reliability. The work of Pélissier et al. [3] described in detail the harvesting techniques as well as technical variations, particularly island flap harvesting and adaptations allowing pedicle lengthening. These advances have enabled the extension of its indications to more complex facial reconstructions.

According to our observations, the use of the submental flap for reconstruction of a cheek defect following excision of a basal cell carcinoma provided satisfactory aesthetic and functional outcomes. These findings are consistent with data reported in the literature, which indicate good tissue integration due to the similarity in skin texture and color between the donor and recipient sites [2,4].

Several studies have highlighted the advantages of this flap compared with other reconstructive techniques. Ferrari et al. [5] notably reported great versatility, with the possibility of adapting the size and shape of the flap according to the defect to be reconstructed. Furthermore, Lee et al. [6] demonstrated low donor-site morbidity, with scars generally being discreet and well tolerated by patients.

Regarding functional outcomes, Liu et al. [4] and Xuwei et al. [7] demonstrated satisfactory recovery of essential functions, such as swallowing, phonation, and mouth opening, particularly in intraoral reconstructions. Although our case involved a cutaneous localization at the cheek level, we also observed favorable healing without any significant functional deficit.

The reliability of the submental flap is further confirmed by the high survival rates reported in the literature. Hayden [8] described a survival rate close to 100%, even in complex settings, thereby demonstrating the robustness of its vascularization. Similarly, Rahpeyma et al. [9] reported complete flap survival in their series, without major complications.

Complications generally remain uncommon. The main reported complications include hematomas, infections, and, more rarely, nerve injuries, particularly involving the marginal mandibular branch. Uppin et al. [10] reported several minor complications, emphasizing the importance of meticulous dissection and thorough anatomical knowledge to minimize these risks. In our case, no postoperative complications were observed, which is consistent with the findings reported in published series.

However, certain limitations should be considered. The use of the submental flap may be questionable in patients presenting with suspicious cervical lymphadenopathy because of the potential risk of tumor spread along the pedicle. Rigorous preoperative assessment is therefore essential to identify patients suitable for this technique [2].

Finally, compared with free microvascular flaps, the submental flap offers the advantages of a simpler technique, shorter operative time, and lower morbidity, making it a particularly attractive alternative in resource-limited settings or for fragile patients.

Thus, our observation confirms that the submental flap constitutes an effective, reliable, and reproducible reconstructive option in the management of facial defects, providing satisfactory aesthetic and functional outcomes, in accordance with the literature.

## Conclusions

The submental flap is a complex and versatile surgical procedure that offers significant advantages in facial reconstruction and restoration of function. Advances in surgical techniques, combined with innovations in medical technologies, have improved both precision and aesthetic outcomes. However, challenges remain, particularly regarding postoperative complication management and appropriate patient selection. Continued research and development are essential to refine and expand the applications of the submental flap.

Ultimately, this work contributes significantly to the ongoing understanding and improvement of the submental flap procedure. The knowledge gained through this research may serve as a strong foundation for future developments in reconstructive surgery, paving the way for more effective and personalized interventions for patients requiring facial reconstruction.
